# Indian pharmaceutical patent prosecution: The changing role of Section 3(d)

**DOI:** 10.1371/journal.pone.0194714

**Published:** 2018-04-02

**Authors:** Bhaven N. Sampat, Kenneth C. Shadlen

**Affiliations:** 1 Department of Health Policy and Management, Mailman School of Public Health, Columbia University, New York, NY, United States of America; 2 NBER, Cambridge, MA, United States of America; 3 Department of International Development, London School of Economics and Political Science, London, United States of America; Iowa State University, UNITED STATES

## Abstract

India, like many developing countries, only recently began to grant pharmaceutical product patents. Indian patent law includes a provision, Section 3(d), which tries to limit grant of “secondary” pharmaceutical patents, i.e. patents on new forms of existing molecules and drugs. Previous research suggests the provision was rarely used against secondary applications in the years immediately following its enactment, and where it was, was redundant to other aspects of the patent law, raising concerns that 3(d) was being under-utilized by the Indian Patent Office. This paper uses a novel data source, the patent office’s first examination reports, to examine changes in the use of the provision. We find a sharp increase over time in the use of Section 3(d), including on the main claims of patent applications, though it continues to be used in conjunction with other types of objections to patentability. More surprisingly, see a sharp increase in the use of the provision against primary patent applications, contrary to its intent, raising concerns about potential over-utilization.

## Introduction

India began to allow pharmaceutical products to become patented in 2005, in compliance with the country’s obligations under the World Trade Organization’s Agreement on Trade-Related Aspects of Intellectual Property Rights (TRIPS). In doing so, the Indian government inserted a controversial provision into the patent law, Section 3(d), which tries to limit the grant of “secondary” pharmaceutical patents, i.e. patents on new forms of existing molecules and drugs.

Section 3(d) has been the source of considerable conflict. One prominent case that brought the world’s attention to 3(d) was the Indian Patent Office’s (IPO) decision to reject a secondary patent on Novartis’ cancer drug “Gleevec” (imatinib mesylate), a decision that cited Section 3(d) as one of the grounds for rejection. Novartis challenged the constitutionality of Section 3(d) and appealed the IPO’s decision, actions that in turn inspired health activists to embark on a campaign against Novartis and in support of the provision. The legality of 3(d) was upheld, and the decision to reject the Gleevec patent was confirmed by the Intellectual Property Appellate Board in 2009 and then, ultimately, the Indian Supreme Court in 2013 [1,2].

Debates and controversies over 3(d) have not been limited to this one drug. The provision has triggered heated and polarized views on pharmaceutical patents in India, and more broadly in developing countries adopting pharmaceutical patents in compliance with TRIPS. On the one hand, many legal scholars, civil society groups, and international organizations have lauded India’s policy choice, citing 3(d) as a prominent example of a country complying with its international obligations but doing so in a way that can preserve generic competition. In that spirit India’s Section 3(d) is commonly held out as model to follow, and other countries where pharmaceutical patenting is also new are encouraged to act similarly [3–5]. On the other hand, many foreign governments and the transnational pharmaceutical industry regard 3(d) with disdain. The US Government routinely cites 3(d) as among the reasons for including India on the “Priority Watch List” in the United States Trade Representative’s annual Special 301 Report [6], for example, and the provision has drawn repeated criticism from international drug firms and their representatives [7,8]. The concern that 3(d) makes it difficult to get a patent in India is widespread in the scholarly literature as well [9,10]. However, these analyses did not look specifically at the role of 3(d) itself, but measures of patent protection on molecules which could be influenced by other factors, including the timing of TRIPS implementation in India.

Previous empirical analyses that did look directly at 3(d) found little independent role of 3(d) in shaping Indian pharmaceutical patent outcomes [11,12]. Specifically, these analyses found that the provision was involved in a relatively small number of cases, and, where it was, it was almost always used together with other more conventional reasons for rejecting patent applications, such as lack of novelty or inventive step. However, the previous analyses were based on pharmaceutical application filings and examination decisions in the early years after the introduction of pharmaceutical patenting in India. It is possible that the role of 3(d) has changed over time, given implementation lags and updated guidance to IPO examiners. Moreover, where 3(d) and other grounds for rejecting patents were employed, previous analyses were unable to untangle which were the main reasons for rejection.

This paper uses new micro-level prosecution data to examine changes over time in 3(d) and to assess the independent role of this provision. While analyses of patent prosecution process are now common for applications filed at the United States Patent and Trademark Office. [13,14], there are few empirical analyses of developing country patent prosecution. This is particularly crucial for analyzing patent prosecution in the context of TRIPS, given concerns that developing countries’ practices may differ substantially from their laws on the books [15].

As we seek to understand how the patent office functions and, specifically, the role of Section 3(d) in patent prosecution processes, we focus not just on the IPO’s final decisions, but also examiners’ initial reports, as well as the exchanges that occur between applicants and the patent office following issuance of the initial reports. Focusing on the first examination reports (FERs) provides a fuller picture of the role of 3(d) in patent prosecution, allowing us to understand how 3(d) is used by examiners and how applicants respond to 3(d) objections that are raised in the course of examination. Another novel aspect of our approach is that we examine the role of 3(d) and other substantive grounds for rejection in targeting the first claim of patent applications. This allows us to assess whether 3(d) struck the core of the application, and whether it did so on its own or in conjunction with other aspects of patent law. Analyses of FERs, which we see relatively early in the prosecution process, also avoids the problem of censoring which complicates assessment of grant rates. This is particularly important for examining changes over time. There is a trade-off, however, as we do not see final decisions in most cases, as we discuss more below.

We find a sharp increase in the prevalence of 3(d) in FERs over time, including on applications’ most important claims. However, 3(d) rarely works alone: it continues to be invoked along with other more conventional objections, even when it is used on an application’s main claim. While the provision does appear to make obtaining a patent more difficult and the prosecution process longer, it is hard to know whether this is due to the independent effects of 3(d), the types of applications that draw 3(d) objections, or the types of examiners that invoke 3(d). Surprisingly, we also find evidence that 3(d) is more commonly used for primary patents than secondary patents, suggesting that it is functioning differently than intended.

The paper has 5 sections. Section 2 provides brief background and context on the introduction of pharmaceutical patents in India and Section 3(d), along with an overview of the patent prosecution process. Section 3 describes the data and empirical approach. Section 4 presents results, examining the changing utilization of 3(d) over time in FERs, the relationship between 3(d) and novelty and inventive step, the association between 3(d) in FERs and final outcomes, and the use of 3(d) on primary vs. secondary patent applications. Section 5 presents discussion of the main findings, indicates directions for future research, and links research on the role of Section 3(d) to broader issues regarding the implications of pharmaceutical patents in India for access to medicines in poor countries in the context of TRIPS.

## TRIPS, pharmaceutical patents, and Section 3(d)

The World Trade Organization’s (WTO) Agreement on Trade-Related Aspects of Intellectual Property Rights (TRIPS) requires all countries to grant pharmaceutical patents. With the exception of “Least Developed Countries,” all WTO members that did not already allow pharmaceutical patents as of 1995, when TRIPS went into effect, had until 2005 to begin doing so. During the transition period, from 1995 until the date that a country made pharmaceuticals patentable, TRIPS required members to receive and hold applications in a “mailbox.” Thus, if in a given country pharmaceutical patents were to become available as of 1999, from 1995 to 1999 the country would accept applications in the mailbox, and these would be examined as of 1999, along with other applications received from that date onwards.

India was one of the countries that most resisted TRIPS during the Uruguay Round trade negotiations of the late 1980s and early 1990s. India opposed the inclusion of rules on countries’ intellectual property policies and practices in the international trade regime, and once the “trade-IP” linkage was established and TRIPS negotiations began, India adamantly resisted the subsequent obligation that all countries allow pharmaceuticals to be patented [16–20]. Although process patents were available in India, product patents had been prohibited since 1970. The absence of patent protection in India coincided with substantial development of the local pharmaceutical sector, and TRIPS was thus perceived as a serious threat [19,21,22]. Perhaps not surprisingly, when forced to allow drug patents but allowed a transition period before doing so, India waited until 2005 to make pharmaceutical products patentable, the maximum period allowed. Indeed, India is one of the only countries to use the full transition period and delay pharmaceutical patenting until 2005. And, also in grudging compliance with the country’s new international obligations, as of 1999 India also began receiving applications in a mailbox, to be examined as of 2005 when the product patent regime was in operation.

In 2005, at the point of introducing the final amendments to the Patents Act to allow for pharmaceutical patents, the Indian government included Section 3(d), a provision that establishes a high barrier for secondary patents. Specifically, 3(d) stipulates that many secondary patents are not considered as inventions, and thus not eligible for patents, unless the applicants demonstrate that these have greater efficacy:

The following are not inventions within the meaning of this Act… The mere discovery of a new form of a known substance which does not result in the enhancement of the known efficacy of that substance or the mere discovery of any new property or new use for a known substance or the mere use of a known process, machine or apparatus unless such known process results in a new product or employs at least one new reactant. For the purposes of this clause, esters, ethers, polymorphs, metabolites, pure form, particle size, isomers, mixtures of isomers, complexes, combinations, and other derivatives of known substance shall be considered to be the same substance, unless they differ significantly in properties with regard to efficacy.

Section 3(d) was implemented explicitly to address concerns that additional patents on existing substances could be used to extend market exclusivity and delay generic competition. Basheer and Reddy [23] report that the Minister of Commerce at the time the patent law was being finalized introduced 3(d) to prevent “ever-greening” [24–26]. While some actors sought a more restrictive approach, for example prohibiting all secondary patents, the designers of 3(d) sought a middle ground that would allow patents on modified forms of existing compounds so long as they demonstrated improvements (“efficacy”) over the earlier, known substance. This intermediate position was subsequently supported by a government-established committee that was asked to report on whether India should prohibit patents on all “incremental innovations” [27].

Thus to obtain a pharmaceutical patent in India, not only do applicants have to satisfy traditional criteria that are common across all countries, e.g. novelty and inventive step, but also meet Section 3(d) requirements. As indicated in the introduction, Section 3(d) has received considerable attention, but its effects have tended to be exaggerated by both supporters and critics. We use micro-level data to shed new light on India’s new pharmaceutical patent system and the role of 3(d).

Before proceeding to the data and analyses, a quick review of the Indian pharmaceutical patent prosecution process may be useful. Applicants must request examination by the IPO within 4 years after their application’s international priority date; failure to do so leads to applications being classified as “withdrawn.” When the IPO examines applications, a first examination report is typically issued within six months. FERs range from a few lines to long and detailed documents with extensive discussions of claims. FERs are like “first office actions” in the U.S., which list objections such as novelty and inventive step, as well as other less substantive grounds such as lack of clarity and mistakes in the application. If an applicant does not respond to the FER the application is “abandoned.” When the applicant does respond, amending or eliminating claims, or rebutting the objections raised by examiners, the IPO then issues a second report and, typically, invites the applicant to a hearing. If the applicant overcomes these objections the patent is granted. If, however, the applicant stops pursuing the application after initially having replied to the FER, for example the applicant does not respond to the IPO’s second report or does not attend the hearing, or does take these steps but is unable to convince the patent office of the merits of the case, the application is refused.

## Data and empirical approach

We started with a set of pharmaceutical applications that were filed globally via the Patent Cooperation Treaty (PCT), both to focus on relatively important applications and to allow for comparability of Indian outcomes to those in other jurisdictions. Accordingly, we began with the September 2015 version of the OECD Triadic Patent Families database, which covers all applications filed in the European Patent Office, US Patent and Trademark Office, and Japanese Patent Office. Using this database, we focused on all “pharmaceutical” applications with priority years (first global filing years) 2000–2012. We then collected information from the WIPO statistics database on all Indian national stage applications; since at the time we collected the data the Indian data were truncated in 2012, we focus the subset with Indian applications filed through 2011. For tractability, we focus on applications with priority PCT month July. This resulted in 1,964 PCT applications, mapping to 1,993 Indian national stage applications. (Since India took full advantage of the transitional period to introduce pharmaceutical patents that was allowed by TRIPS, as explained above, the applications in our dataset that were filed in India from 2000–2005 were held in a “mailbox” until examination commenced in 2005.)

We collected Indian outcomes on all 2000–2011 applications from the Indian patent database as of May 2017. We record five mutually exclusive categories: applications can be granted, pending (still waiting final determination), withdrawn before examination, abandoned after a first examination report issued, or refused. As explained above, if an applicant pursues the application after receiving the FER but is unsuccessful in overcoming the objections raised, the application is considered formally refused. We also collect data on duration of prosecution for granted patents.

As explained, a novel contribution of our work is that we analyze the first examination reports issued by the patent office after applications have undergone their first substantive review. For all applications with FERs we determined if the reports included any 3(d) objections, and also whether they included any novelty or inventive step objections. We also determined whether there were 3(d) objections on the first claim, and, for a subset of applications, whether there were novelty or inventive step objections on Claim 1 as well.

While most of our analyses of 3(d) focus on FERs, we also use the full prosecution record of some applications to gain a stronger sense of the role of 3(d). For all applications where there was a 3(d) objection on claim 1 of the FER and a final outcome of refusal, and for a random selection of applications with 3(d) objections on claim 1 that ultimately were granted by the patent office, we read through the correspondence between applicants and the patent office (e.g. replies to FERs, subsequent examination reports, controller’s reports) to understand how applicants respond to 3(d) objections and the role of 3(d) throughout the prosecution process.

To examine the different roles of 3(d) for different types of applications, we code each of the applications in our sample as to whether they claim a new compound (“primary” patent applications) or, alternatively, a modified form, composition, or use of an existing compound (“secondary” patent applications) using the coding scheme from previous research [11,12]. The claims coding also revealed a handful of pure process applications. After dropping these we were left with 1853 applications. S1 File provides details on data construction, as well as links to the datasets, our coding of patent application type, outcomes, and prosecution histories, and computer code to fully reproduce the results below.

## Results

We use these data to address the following questions:

1. How has the use of 3(d) by examiners in FERs changed over time?
2. How much overlap is there between 3(d) and novelty/inventive step objections in FERs?
3. How does the inclusion of 3(d) objections in FERs, alone or in conjunction with novelty or inventive step, correlate with different outcomes?
4. What kinds of patent applications draw 3(d) objections in FERs?

### The changing role of 3(d) over time

To examine the role of 3(d) over time, we focused on applications that have FERs. The share of applications with an FER drops over time (for example, from about 78 percent in the 2001–2004 period to 52 percent in the 2008–2011 period). This is not surprising, as examination has not yet begun on a larger share of more recent applications. We were able to locate FERs for nearly all abandoned, granted, and refused applications (as well as a third of the pending applications, where examination has begun but not yet concluded), yielding 1,283 FERs. Overall, 37 percent of the applications with FERs are granted, 45 percent abandoned, 5 percent refused, and 13 percent pending.

**Fig 1:**
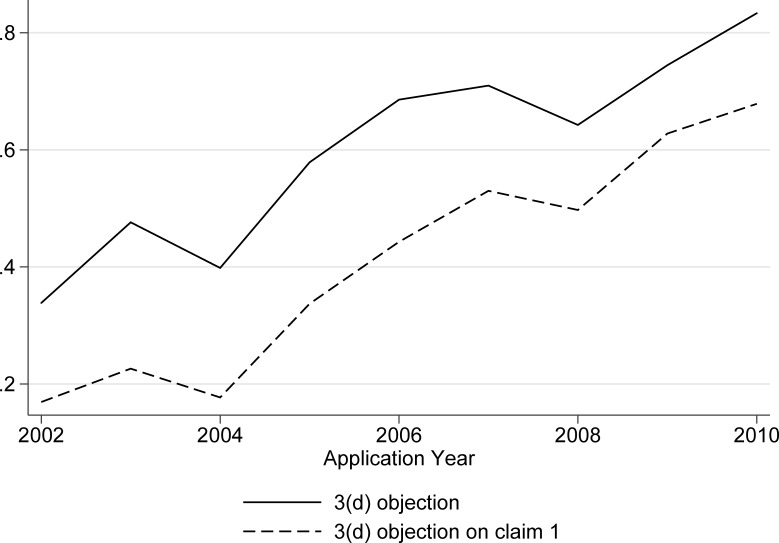
Share of applications with 3(d) objections in first examination report (FER). Based on 1283 applications with a first examination report.

The solid line in Fig 1 shows the share of applications with an FER with any 3(d) objection, by application year. The sharp increase over time, from less than 40 percent of the early applications to more than 80 percent of the most recent applications, demonstrates an increased utilization of 3(d) by Indian patent examiners. While previous research, based on even earlier sets of applications, revealed a low incidence of 3(d) [11,12,28,29], this is clearly no longer the case.

We also examined the extent to which 3(d) targeted the first claim of the application. Patent applications include “independent” and “dependent” claims, with the latter following and based on the former. Claim 1 is, by definition, an independent claim, and typically the broadest and most important claim [30–32]. One challenge to studying the incidence of 3(d) objections on claim 1 is that in many instances the FERs do not indicate specific claims, e.g. the objection is simply that “claims fail to satisfy” Section 3(d). The share of ambiguous FERs of this sort, where Section 3(d) is raised but in a way that does not indicate specific claims, declines sharply, from about 39 percent in 2001–2004 to 14 percent in 2008–2011. This trend reveals greater sharpness and focus in the patent office’s use of 3(d) and examination practices more generally.

The bottom line of Fig 1 shows that the share of applications where there are specific 3(d) objections on claim 1 is also rising sharply over time. By the end of the period under study, in excess of 60 percent of the FERs include 3(d) objections on the first claim. Not only is 3(d) increasing over time, but it is typically, and indeed increasingly, striking at the core of the patent application.

Taken together, we find (1) a sharp growth in the use of 3(d) over time, (2) that it tends to target first claim of the application, and increasingly so. Unlike previous work suggesting underutilization of 3(d), here we find evidence that IPO is using 3(d) to try to reject almost everything, in the first examination report at least.

### Is 3(d) redundant?

The data presented so far suggest that 3(d) is a major way in which the Indian Patent Office tries to limit patent grants, and increasingly used over time. This is consistent with concerns that 3(d) makes it harder to obtain patents in India than other jurisdictions (as it was meant to do). However, one wrinkle is that we do not know what work is being done by 3(d) itself. Examiners may also be objecting to patents on other, more traditional grounds, such as lack of novelty or inventive step. Indeed, previous research has suggested just that, that Section 3(d) was rarely used alone, but rather in conjunction with other ways of rejecting applications [11,12]. We explore this here too, both overall and for the main claim. Specifically, we also identified novelty and inventive step objections on the 427 FERs for applications filed between 2006 and 2007. We focused on applications for which there were electronic FERs, dropping 9, leaving 427. An advantage of looking at this time period is that the applications are more likely to have FERs (86 percent do) and the FERs are more likely to have clearer delineation of specific objections on specific claims.

**Fig 2:**
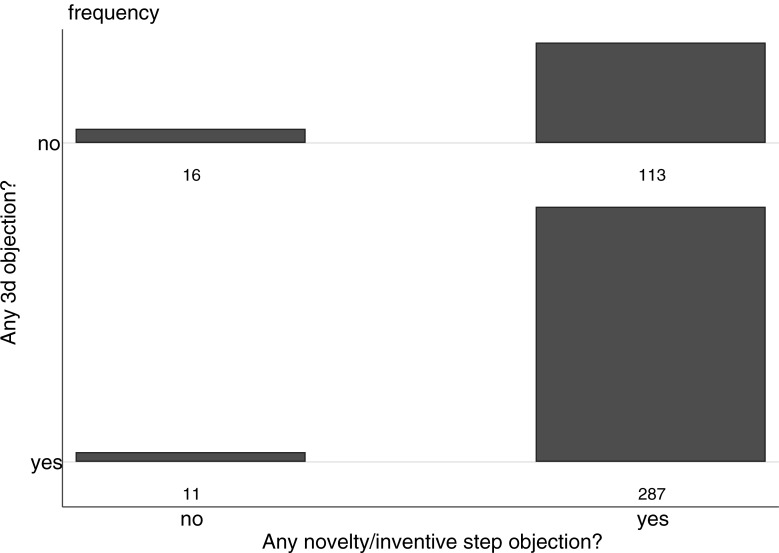
Distribution of objections in first examination report for 2006–7 applications. Based on 427 applications filed in 2006 and 2007 with a first examination report.

First, we looked for whether applications with any 3(d) objections had other sorts of objections. Fig 2 shows the distribution:

The majority of FERs (67 percent) cite both 3(d) and novelty/inventive step, and only 4 percent cite neither. At the application level, Section 3(d) rarely is used on its own: of the FERs with 3(d) objections the vast majority of these applications (96 percent) also draw novelty or inventive step objections.

**Fig 3:**
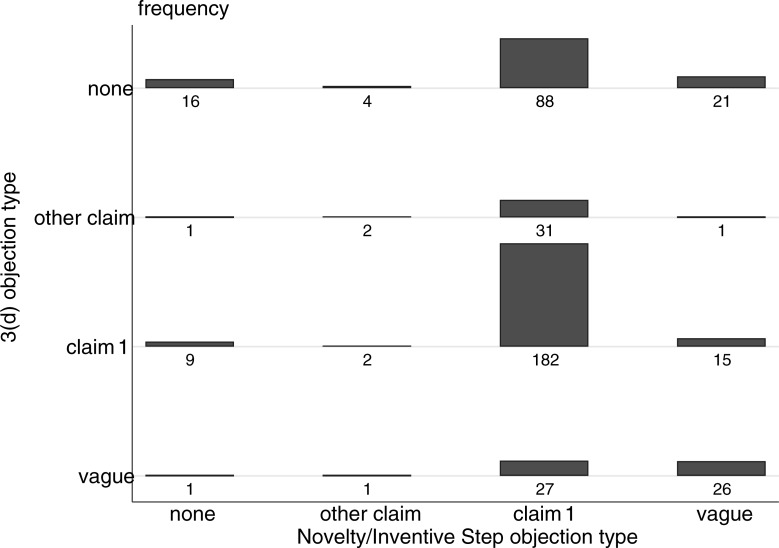
Distribution of objections in first examination reports for 2006–2007 applications. Based on 427 applications filed in 2006 and 2007 with a first examination report.

Although this overlap suggests redundancy between 3(d) and more traditional patentability criteria, it is possible that, within a given application, these different criteria are being invoked against different claims. Might 3(d) be used for important claims independently of novelty and inventive step? To investigate this, we examined overlap between 3(d) and novelty/inventive step at the level of claim 1. We use the same four categories as above to classify each FER (no objection, objection not directed at claim 1, objection including claim 1, objection presented without specific claims), now for 3(d) as well as novelty/inventive step. Fig 3 shows the distribution:

Where 3(d) is raised against claim 1, the same claim is also subject to a novelty or inventive step objection more than 88 percent of the time (and for another 7 percent of these novelty or inventive step objections are raised, but the language of the FERs does not allow us to know for certainty if examiners had claim 1 in mind). Only 4 percent of the time when 3(d) was raised against claim 1 were there no novelty or inventive step objections in the FERs at all. The relationships are less sharp in the other direction. Where novelty or inventive step are raised against claim 1, the same claim is also subject to a 3(d) objection 56 percent of the time (and another 8 percent where 3(d) was raised without specifying the claims), while for 27 percent of the applications where conventional patentability criteria were raised against the first claim, 3(d) was not mentioned.

That the IPO tends to pair 3(d) objections with those based on conventional patenting criteria makes it difficult to know whether 3(d) per se is making examination stricter (e.g. by getting patent examiners to think more carefully about novelty and inventive step), or whether more rigorous examination is leading to more 3(d) and also other types of rejections. This is difficult to know without some source of random variation in 3(d), which we have not been able to identify. However, below we present evidence on associations between 3(d) and final outcomes that provide a sense of whether applications with and without 3(d) objections in first examination reports fare differently.

### The association between 3(d) objections in FERs and final outcomes

The finding that 3(d) objections are present in the vast majority of FERs does not mean that these applications are all rejected (or, if rejected, on 3(d) grounds). As in the US, where the first office action is almost always a “non-final rejection,” in India applicants can and do rebut 3(d) and other objections, or amend claims to address them. If they do so successfully they obtain patent protection.

**Fig 4:**
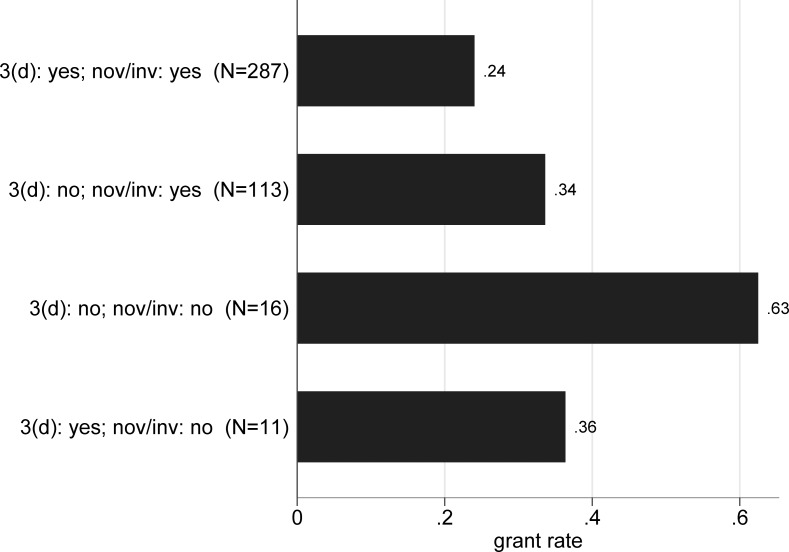
Grant rates for 2006–2007 applications by type of objection, broad. Based on 427 applications filed in 2006 and 2007 with a first examination report.

How often do applications with 3(d) objections get granted? Fig 4 shows that applications with both 3(d) and novelty/inventive step objections have a grant rate of 24 percent, compared to 63 percent for the small number of applications that have neither 3(d) nor novelty/inventive step objections (p < .01). The grant rate for applications with novelty/inventive step objections alone (34 percent) is about 10 percentage points higher than those with novelty/inventive step and 3(d) objections (p = .051).

**Fig 5:**
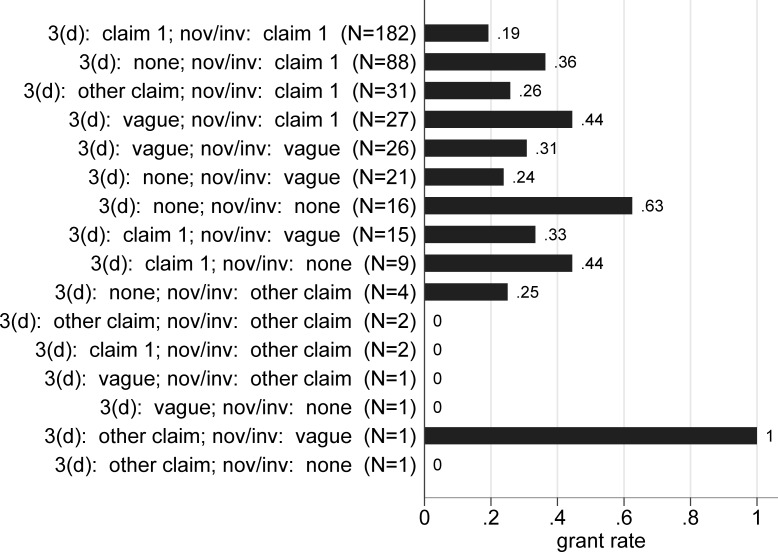
Grant rates for 2006–2007 applications by type of objection, detailed. Based on 427 applications filed in 2006 and 2007 with a first examination report.

Fig 5 shows the same information for the detailed claim level objection grounds, sorted in descending order of frequency. Compared to applications with only novelty/inventive step specifically on claim 1, where the grant rate is 36 percent, those with both novelty/inventive step and 3(d) objections on claim 1 have a much lower grant rate of 19 percent (p < .01).

**Fig 6:**
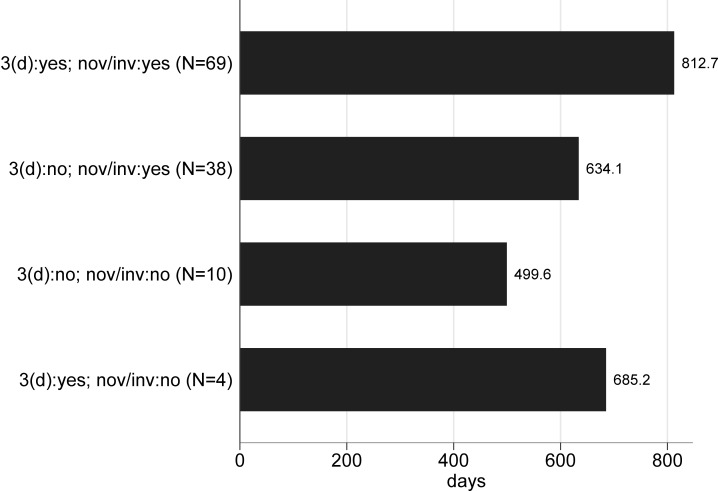
Application-grant lags. Based on grants from 2006–7 filings.

We also considered, for granted patents, whether 3(d) objections (anywhere in the FER) add time to the process. To do so, for granted patents we collected data on the timing from FER to certificate of issue. Fig 6 shows the mean grant lag by type of objections in the FER:

Overall, applications with 3(d) objections that are eventually granted take about 200 more days to be granted than applications where FERs did not mention 3(d) (p = .01). And applications with just novelty/inventive step objections in initial review that end up granted proceed to this outcome on average 178 days more quickly than when they also have 3(d) objections (p = .51).

These results suggest that adding 3(d) to the mix is associated with a more difficult prosecution process (more likely to get rejected, or more likely to abandon the application), and, conditional on getting a patent, dealing with 3(d) is associated with longer lags. But, as indicated above, it is hard to know whether this is because of 3(d) per se. It is also possible that the types of applications that attract 3(d) are less likely to be granted or would be subject to more rigorous examination anyhow, for reasons that we are unable to observe, or that examiners who use 3(d) are more rigorous.

Still another possibility is that early use of 3(d) creates opportunities for the patent office to probe deeper about whether applications meet even standard patentability grounds. There is some evidence of this from the prosecution record of the applications that were ultimately refused. Recall that applications are classified as “refused” only after the applicant has responded to the FER (otherwise they are classified as “abandoned”). These applications also then ordinarily feature a further examination report by the IPO that includes an invitation for a hearing and, ultimately, a Controller’s Decision with a final outcome. We reviewed the full prosecution histories of 20 of the 22 applications in our dataset that had 3(d) objections on claim 1 in their FERs and a final patent office decision of refusal. (For two of these applications the relevant prosecution materials beyond FERs were not available.) In six of these the post-FER prosecution process appeared to feature rigorous application of standard patentability criteria: in three instances examiners’ 3(d) objections were successfully overcome, but the application was still rejected on grounds of lacking novelty or inventive step, and in another three instances novelty and inventive step objections were not raised in the FERs but were cited as grounds for final refusal (in one case solely, without 3(d)). By contrast, in three other instances the FERs cited novelty or inventive step but these were no longer mentioned as grounds for refusal in the final patent office decisions. In the remaining 11 applications, both 3(d) and standard patentability criteria were used the same ways in FERs and final decisions.

### What kinds of applications get 3(d) objections?

Previous analyses of 3(d) have focused mainly on its effects on secondary patent applications, which is natural since these are the applications it was meant to target. Together with the results (above) on the growth of 3(d) objections, prominent cases of 3(d) being used against primary patents (including, in a preliminary ruling, sobusfovir)—those covering drugs’ original molecules–raise the question of whether it is being used more expansively.

Here we return to the full sample of applications with FERs (not just 2006–07), and we use our coding of whether the applications are primary or secondary. As noted above, and discussed in more detail elsewhere [11,12] we categorize as “primary” applications those that include at least one claim on a new compound. Secondary applications include those on polymorphs and crystal forms, enantiomers and isomers, salts, metabolites and derivatives, and other modified forms, compositions, or uses of an existing compound that do not also have a new compound claim. (See discussion in S1 File for more detail.)

**Fig 7:**
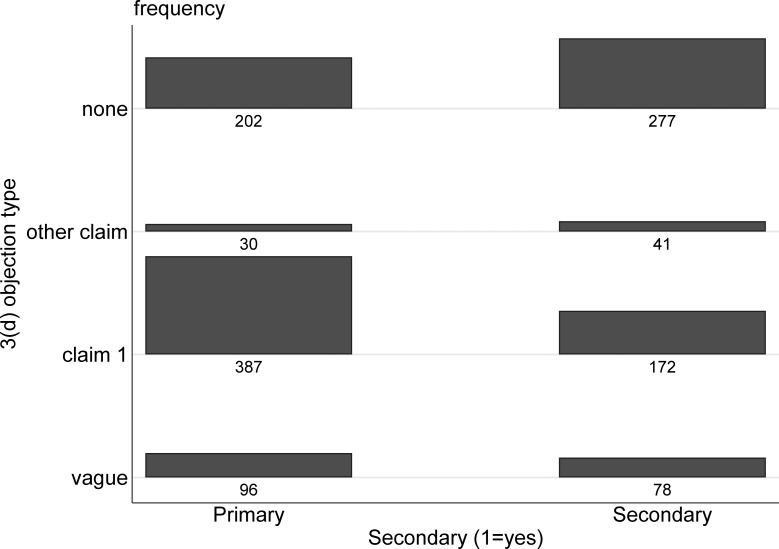
3(d) objections by application type. Based on 1283 applications with a first examination report.

Overall about 56 percent of the applications in our sample are primary, and 44 percent secondary. Yet as Fig 7 shows applications for primary patents account for a disproportionate share, 69 percent, of the applications drawing 3(d) objections on the first claim. Indeed, while 30 percent of secondary applications drew 3(d) objections on the first claim, 54 percent of the primary applications did (p < .01).

**Fig 8:**
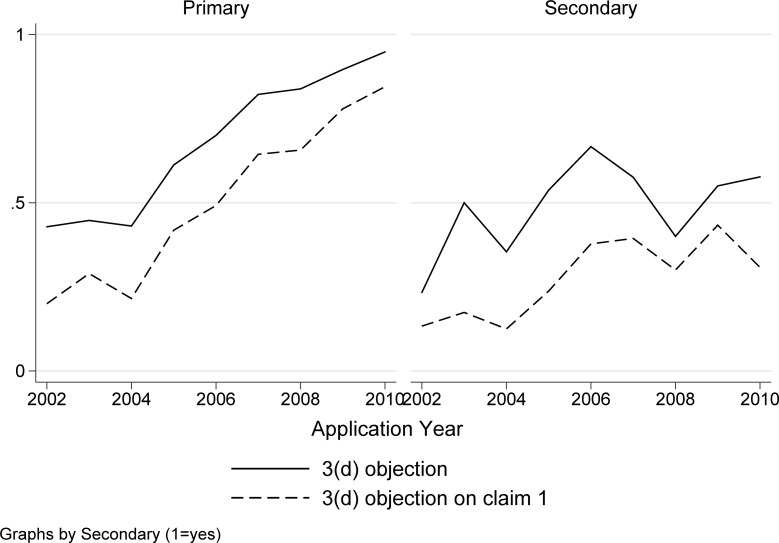
3(d) objections by application type, over time. Panel (a) Primary applications; Panel (b) Secondary applications. Based on 1283 applications with a first examination report.

Moreover, Fig 8 shows that the share of primary applications with 3(d) objections and 3(d) objections on claim 1, is increasing over time, with a less pronounced trend for secondary applications.

The use of 3(d) on primary applications is surprising. To explore this further we reviewed a random sample of 20 granted patents where 3(d) objections on claim 1 had been raised in the FERs, again reviewing the entire prosecution history. One way that applicants overcome 3(d) objections is by presenting comparative data to satisfy 3(d)’s requirement that new forms of known substances demonstrate increased efficacy. Or, in the absence of such data, applicants amend or remove claims such that 3(d) is no longer relevant. In many instances, however, applicants reject the relevance of this provision altogether. In 16 of the 20 cases, applicants insisted (successfully, as the patents were granted) that their compounds were new and therefore not subject to 3(d).

In doing so, applicants commonly reprimand the IPO for inappropriately applying 3(d), reminding the patent office that this provision was intended to prevent patents on new forms of known drugs, and not as a block on new compounds altogether. As an illustration, consider the reply by Eli Lilly on the FER for application 2252/MUMNP/2009, insisting that its claimed invention is novel and accusing the IPO of abusing 3(d):

“Section 3(d) was designed to make a higher bar of innovation for patentability of new salts, esters, etc. of know substances (e.g. pharmaceuticals), to avoid trivial, inconsequential alterations being made to the FORM of such substances and thus extending market exclusivity of known substances. It is NOT meant to create a higher bar for new substances by deeming all new compounds to be merely derivatives of known compounds…. It is submitted that Section 3(d) is intended to block patenting by individuals who would make trivial changes to the form of a known compound, such as making a different salt, ester or other prodrug, which would be expected to retain the original activity of the parent compound. This provision was NOT intended to be a secondary, higher bar to patentability of novel compounds over and above inventive step…. The present improper use of 3(d) attempts to define every new compound as merely a derivative of some know structural chemical core thereby barring patentability, despite the compound’s novelty and inventiveness in the unpredictable chemical arts” (pp. 2–3, emphasis in original).

## Discussion and conclusion

The data reveal substantial increases in the use of 3(d) over time in FERs, overall and with specific regard to the main claim. Clearly, the IPO is relying extensively on 3(d) to raise a higher barrier for obtaining pharmaceutical patents.

While the increased reliance on 3(d) may reflect characteristics of the applications filed in India, this may also reflect explicit policy. In the initial years of India’s new pharmaceutical patent regime, many observers asserted that, notwithstanding the high-profile Gleevec case, 3(d) tended to be under-utilized [11,28,29]. The association representing India’s leading pharmaceutical firms published a report, authored by the former director of intellectual property in the Ministry of Commerce calling for more aggressive application of Section 3(d), for example, and subsequently worked with the IPO to revise the examination guidelines to that effect [28]. And the defense of 3(d) provided by the Appellate Board and then the Supreme Court [1,2] may have contributed to this too, by giving examiners greater confidence to use this provision. It is difficult to ascertain the effects of constituent pressures, revised guidelines, and legal support, though it is reasonable to believe they have contributed to the increased use of 3(d).

But Section 3(d) is rarely used alone. Even when 3(d) is invoked as a reason why a patent should not be granted, it is rarely invoked as the only reason. Examiners also use other, traditional, grounds to deny patents, such as lack of novelty or inventive step. Previous work, at the application level, suggested that this was common [11,12], and the current findings, based on FERs, are consistent with that research: looking at applications filed in 2006–07 for which we could obtain FERs, we find that when 3(d) objections are raised, in nearly all (94 percent) instances so too are objections based on lack of novelty or inventive step.

Overlap between 3(d) and other patentability criteria at the application level does not necessarily imply redundancy in use, as different provisions of the patent law may be applied to different claims within a single application. Researching the use of 3(d) and other provisions at the claims level is difficult, on account of the quality of FERs. In the initial years of patent examination FERs tended to be too vague, simply indicating that “claims” do not satisfy the tests of 3(d) or other aspects of the patent law, without indicating which claims a given objection was referring to. Looking at a set of applications during the time period when FERs tended to be more specific (but early enough so that FERs have been produced), our findings at the claims level are consistent with what we observed at the application level: in nearly all cases of 3(d) being used against the first claim in an application, so too were novelty and inventive step. Most of the time, whether looking at applications as a whole or the first claim, 3(d) objections are accompanied by novelty or inventive step objections as well. Although there is increasing use of 3(d), it is not independent use.

We also examined association between 3(d) and final outcomes. More than half of the applications that draw 3(d) objections at first examination are ultimately granted. However, grants are less likely if a 3(d) objection is raised in the FER than otherwise, and the grant lag is longer when 3(d) is in the FER.

For abandoned applications, where applicants do not reply to the FER, it is not possible to know how 3(d) affected the final outcome relative to other factors leading applicants to decide not to pursue the patent. For refused applications, however, we can start to understand the role of 3(d) by reviewing the post-FER prosecution record. In contrast to abandoned applications, refused applications (and granted patents, as discussed below) ordinarily feature a reply to the examination report by the applicant and, in turn, a further report by the IPO that includes an invitation for a hearing and, ultimately, a Controller’s Decision with a final outcome. Our review of the full prosecution processes for the refused applications in our dataset where the FERs included 3(d) objections on the first claim revealed rigor throughout the examination process in the use of standard patentability criteria. In the case of applications where 3(d) was initially invoked in the FER but the application was refused on other grounds, for example, the initial use of 3(d) early in the examination process may have created an opportunity for examiners to discover or consider in depth other problems with the application that were not fully appreciated in the initial examination. In sum, patent applications drawing 3(d) objections in their FERs are less likely to be granted and, even when granted, experience a more complicated path to grant on account of 3(d)’s involvement.

Our finding that 3(d) is invoked against the main claims of primary applications is surprising. Section 3(d) demands that “new forms of known substances” demonstrate increased “efficacy” in order to be patented. Applying Section 3(d) thus entails two steps: first, a decision has to be made as to whether the claimed invention is subject to this rule (i.e. if it is derived from a known substance), and, if so, a second decision has to be made as to the efficacy of the claimed invention relative to the known substance. Much of the analysis and debate over this provision has regarded the first step, of determining what efficacy means, how much increased efficacy is enough, relative to what reference point, and how to demonstrate this [23,33–38]. But prior to decisions about efficacy is the first order question about whether the claimed invention is a “new form of a known substance” and subject to 3(d) at all. One would expect primary patents to clear the hurdle set by this first order question. Given the rationale behind introducing Section 3(d) in the first place, to prevent the accumulation of secondary patents that could extend periods of exclusivity, and the associated debates over whether India should bar all secondary patents or only those that fail to demonstrate increased “efficacy” [27,39], it is reasonable to expect primary patents to fall largely out of the purview of this provision. For that reason, we have previously argued that if 3(d) is implemented as intended, new drugs are likely to receive patents in India–but only one patent [40].

But 3(d) may be functioning differently than expected. Section 3(d) is being invoked commonly by examiners against applications for primary patents. Even if many of these patents end up granted, the incidence of 3(d) in FERs against primary applications seems surprising. This result suggests 3(d) may be used in a way that is different from what it was designed to accomplish.

Understanding how and why 3(d) is used against what we are calling primary patents is important, and warrants more research. All applications are subject to 3(d) assessment: if examiners determine that the claimed invention is “new” then 3(d) is not involved, but if they conclude that the claimed invention is derived from a compound that is already known, then the application is subject to the provision (and will be evaluated against its efficacy standard). In the first step of this process, an examiner**’s** assessments of what constitutes a new compound vs. a new form of a known compound is what matters. This distinction is not always clear cut. By one perspective, once a new class of compounds is created, most compounds that follow and have similar basic chemical structure are “derivatives” and therefore subject to 3(d). These would be primary patents in typical categorization (and in our coding). If these are subject to 3(d) then this provision could have broader effects beyond secondary patents as traditionally defined. To be sure, there must (at least hypothetically) be genuinely “new” compounds that are not be vulnerable to 3(d), but it is uncertain where exactly the line between non-vulnerable and vulnerable primary patents might be [23].

The possibility of 3(d) being used to block primary patents received considerable attention in January 2015, when the IPO used 3(d) to reject a compound patent on sofosbuvir (“Solvadi”), Gilead’s important Hepatitis C treatment. In this instance, the IPO regarded the compound that Gilead sought to patent as being a close derivative of an existing molecule, and consequently rejected the application due to lack of efficacy. Although Gilead eventually prevailed on appeal and received a patent, the events are revealing. During the course of the prosecution, Gilead denied the relevance of 3(d), maintaining that the claimed was new and therefore not subject to questions of efficacy. During the course of the prosecution, Gilead denied the relevance of 3(d) on precisely the same grounds as what we observe in our analyses of 3(d) and primary patents, and also criticized the IPO’s “improper use” of this provision in precisely the same terms cited above [41].

While 3(d) objections against primary patents may not necessarily persist through the prosecution process and these patents may be granted, its use in this way may still create challenges for the drug industry. Convincing the IPO that the claimed invention is not a derivative and therefore that 3(d) is not relevant (or demonstrating therapeutic efficacy where 3(d) is relevant), is not a formality; doing so takes time and resources. It is no surprise, then, that the transnational pharmaceutical industry intensely dislikes Section 3(d). As discussed above, some responses to FERs explicitly assert that 3(d) is being applied too aggressively in patent examination.

While this article has focused on the use of Section 3(d) by examiners in FERs, our research is also suggestive for how the IPO’s use of Section 3(d) may affect outcomes. As explained, it may be that applications that draw 3(d) objections have characteristics that make them less likely to be granted anyway. Or perhaps variation is at the level of examiners themselves, that some are more inclined to invoke 3(d) and that those who use 3(d) tend to be more difficult to satisfy overall. We are unable to know the precise channels with the data and types of variation available.

Theoretically, there are several indirect mechanisms through which 3(d) could be influencing outcomes, in addition to its direct effects. First, invocations of Section 3(d) in FERs may function, in effect, similarly to how “deferred examination” rules are known to function [42], and thus lead to applications that may otherwise be granted to be abandoned or refused. Applicants have to reply to 3(d) objections to obtain a granted patent, and in the course of preparing their replies, or deciding whether to do so, they may determine that it is not worth their time and effort to pursue the patent (often for commercial reasons that may have little to do with the quality of the application and likelihood of grant). Applications of this sort, had they been pursued, may have been granted, but they were not, and therefore end up as non-grants.

The use of Section 3(d) early in the examination process may also generate opportunities for raised scrutiny at subsequent stages of the prosecution process. After an FER cites 3(d), subsequent exchanges between applicants and the IPO may reveal other deficiencies and weaknesses in the claims, deficiencies that were not noticed in the initial examination. The presence of a 3(d) objection in the FER may be associated with more rigorous subsequent examination by the IPO.

More broadly, it is possible that this single provision makes the entire examination process more rigorous. Given the attention that 3(d) has received, as a mechanism to blunt the impact of pharmaceutical patenting, it may signal to examiners to be more rigorous and search a bit harder to find reasons to object to patent applications. After all, the same actors–pharmaceutical patent examiners and patent office controllers–are responsible for applying 3(d) as well as conventional elements of patent law. In contrast to a previous legal literature which emphasizes the emergence of a shared “interpretative culture” among patent offices such that non-meritorious claims come to be seen as patentable [15,43], the presence of Section 3(d) in Indian patent law may lead to more rigorous application of novelty and inventive step. Indeed, some of the novelty and inventive step objections raised by the IPO may not have been noticed or cited, in the FERs or later in the prosecution process, if not for 3(d) making the examination process more rigorous in the first place. Identification of the direct and indirect effects of 3(d) on outcomes is a subject for future research.

Beyond its local significance, analysis of how the Indian pharmaceutical patent system functions has important global implications. The extent to which drug companies can expect patent protection in developing countries will hinge on how 3(d) works, in general, and on its application toward different types of patents. If 3(d) is interpreted to cover traditionally “primary” patent applications as well as secondary ones, it will be difficult to obtain patent protection in India even for new drugs. (By new drugs, we mean drugs approved by regulators as new molecular entities, which typically have at least one patent application we would classify as primary.) If the provision is focused mainly on secondary applications, its impact will be mainly on the number of patents per drug and thus duration of patent protection, rather than on whether new drugs receive any patent protection at all.

These different scenarios also have implications for access to medicines beyond India. Indian pharmaceutical firms have played a central role as provider of affordable, high-quality, generic medicines throughout the developing world [44, 45]. While many of the drugs supplied by Indian firms are older medicines where any patents have long since expired, Indian firms also supply their own versions of more recent drugs that, while patented in some countries, lack protection in India because they pre-date the product patent regime. But now India grants patents: all drugs with post-1995 priority dates are in principle (under TRIPS) eligible for patents in India [40]. India’s ability to continue providing generic drugs to developing countries—and its role as “Pharmacy of the Developing World”—will depend crucially on how broadly Section 3(d) is interpreted and implemented. Specifically, if 3(d) serves as a block on primary patents this will make Indian generic production and distribution of new drugs easier. If, instead, 3(d) is focused on secondary patents, going forward Indian generic production and distribution will not be possible until later in the lifecycle of most new drugs, after their primary patents expire.

## Supporting information

S1 File “Data Appendix.pdf” This file provides information on data construction and code to reproduce the all results in the text. (PDF)
