# Supplementary material for: Indian pharmaceutical patent prosecution: The changing role of Section 3(d)
Source: PLoS One. 2018 Apr 2;13(4):e0194714. doi: 10.1371/journal.pone.0194714 (PMC5880378; doi:10.1371/journal.pone.0194714)
Supplement: S1 File — (PDF) [file pone.0194714.s001.pdf]

Data Appendix: Indian pharmaceutical patent prosecution: The changing role of Section 3(d)

Introduction

This Appendix aims to provide Stata code to fully reproduce all results in the paper, using the data files available from Bhaven Sampat’s Dataverse at <https://dataverse.harvard.edu/dataset.xhtml?persistentId=doi%3A10.7910%2FDVN%2FKNWKIV>.

This is a dynamic Stata document (<https://www.stata.com/new-in-stata/markdown/>), meaning that running the command `dyndoc "code/Data Appendix.md"`, replace after creating the directory structure below and copying the files from Dataverse to the appropriate directories should fully reproduce the results in the paper. (If using the Dataverse files, download the `src` and `byhand` files in original format and place in those directories. Two packages `tabplot` and `scheme_tufte` are required. The output from the `dyndoc` command (reproducing all output in the paper) is written to `Data Appendix.html`.

The `dyndoc` command will run in Stata 15.0 or later versions. For those without Stata 15.0 the code from this document can be pasted into a `.DO` file for any recent version of Stata to reproduce the results as well. We also include CSV version of the final analysis file for those who prefer to analyze that directly, even using different software.

In the “Background and Setup” section below we discuss data sources and preparation. If you are interested only in the final files used for analyses and to produce the figures and other output in the paper, please skip to the “Analysis” section that follows.

Background and setup

If you wish to run the `dyndoc` command to reproduce, create the following directories and populate the `src`, `byhand` and `code` directories in the directories indicated on Dataverse.

Directory	Description
<code>src</code>	raw OECD TPF data
<code>byhand</code>	files coded by hand
<code>processed</code>	final datasets used for analysis
<code>tmp</code>	temporary datafiles generated during processing
<code>code</code>	this data document and/or individual DO files

We began with the OECD Triadic Patent Families (TPF) database (September 2015) which covers patent applications filed to the EPO, the JPO and the USPTO that share the same set of priorities. (The OECD database is a subset of the from the EPO PATSTAT database, with various value added fields. We downloaded the OECD TPF from the public FTP site here: <ftp://prese:Patents@ftp.oecd.org/>. Since this site does not store archival versions, we include the relevant tables in the ZIP file provided above.

Specifically, we used data from three tables from the TPF:

1. 201509_TPF_Core.txt
2. 201509_TPF_PCT.txt
3. 201509_TPF_IPC.txt

The unit of analysis is a patent family, denoted by the variable *family_id*. The analyses focus on applications that met the following criteria:

1. Have main international patent class (IPC) of “A61K” or “C07D” (to focus on pharmaceutical applications)
2. Have priority filing years between 2002 and 2012
3. Have a PCT filing
4. Do not have Derwent patent codes associated with biologic drugs or non-pharmaceutical inventions
5. Have a least one Indian national stage application
6. Have priority month of July

Below, we discuss each of these.

Creating an extract from TPF dataset

We began by converting the three TPF files to Stata format:

```
<<dd_do: noout>>
2
/* Setup: This linewidth helps make the final output
   files look nicer */
4
set linesize 80
6
/* Import the TPF_Core, TPF_IPC, and TPF_PCT files
   and save to temporary directory */
8
foreach i in Core IPC PCT {
10 insheet using ../src/201509_TPF_`i'.txt,
    delimiter("|") clear
    save tmp/TPF_`i', replace
12 }
```

14 <</dd_do>>

Next, we worked with these files to isolate applications with IPC code A61K and/or C07D, priority filing years 2000 to 2012, and a PCT filing:

```
<<dd_do: noout>>
2
  /* Keep the applications with one of the two IPC
    codes anywhere */
4
  use tmp/TPF_IPC, clear
6 keep if index(ipc,"A61K")==1 | index(ipc,"C07D")==1

8 /* Keep unique application identifier */

10 bysort family_id: keep if _n==1
  keep family_id
12
  /* Save these to temporary directory to be used later
    */
14
  save tmp/family_to_ipc, replace
16
  /* Keep the applications with priority years 2000 -
    2012 */
18
  clear
20 use tmp/TPF_Core, clear

22 /* Generate a priority year variable and keep in
    range */

24 qui: tostring first_prio, gen(prio_string)
  gen prioyear = real(substr(prio_string,1,4))
26 keep if prioyear>=2000 & prioyear<=2012

28 /* Merge in matching applications from IPC A61K /
    C07D set */

30 joinby family_id using tmp/family_to_ipc,
  unmatched(master)
  keep if _merge==3
32 drop _merge

34 /* Keep only the applications with a PCT filing */
  keep if count_pct~=.
```

```

36 /* get the PCT number */
38
40 keep family_id first_prio
40 joinby family_id using tmp/TPF_PCT, unmatched(master)
40 assert _merge==3
42
42 /* keep family_id, pct_number, priority_date */
44
44 keep family_id pct_nbr first_prio
46
46 save tmp/TPF_extract, replace
48
48 <</dd_do>>

```

Filtering by DWPI class

We learned from previous research (Sampat and Shadlen 2017) that filtering by IPC class was a good first step, but that many resulting applications were not drug-related, or related to biologic drugs. Since our interest is in small molecule drugs, we filtered further using data from the Derwent World Patent Index.

Specifically, we searched for each PCT application in the Derwent World Patents Index, accessed through Thomson Reuters (now Clarivate):

<https://clarivate.com/products/dwpi-reference-center/>

We downloaded the DWPI manual code for each application, and isolated applications that were drug related (having DWPI category “B”) but not biologic (not having DWPI code B-04E, D05-H, B04-F, B-04G). The resulting applications are in the file `byhand/dwpi_pharma.csv`. About half of the original dataset remains after this filter:

```

<<dd_do: noout>>
2 /* Import the DWPI search results: these are "pharma"
   apps left after DWPI filtering */

4 insheet using byhand/dwpi_pharma.csv, comma names
   clear

6 /* Merge back the TPF file */

8 joinby pct_nbr using tmp/TPF_extract, unmatched(both)
   tab _merge
10 keep if _merge==3
   drop _merge

```

```

12 save tmp/TPF_extract_pharma, replace
14 <</dd_do>>

```

Obtaining Indian national stage applications

The next step was to collect information on national phase application numbers. There are several ways to do this, including searching the Indian Patent Office database <<http://ipindiaservices.gov.in/publicsearch>> or Patent Scope <<https://patentscope.wipo.int/search/en/search.jsf>> databases by hand.

Rather than search by hand, we provided our PCT numbers to WIPO who produced for us corresponding Indian application numbers from the WIPO Statistics Database. We verified this was essentially identical to that which would have been available from searching Patent Scope or Patent Scope as of late-2016, when we conducted the searches. These applications are in the file `byhand/wipo_india.csv`.

We obtained these data from the WIPO Statistics Database. Unfortunately the Indian national data in that file is not complete for PCT applications filed after 2012, so we focus on PCT applications filed until the end of 2011.

```

<<dd_do: noout>>
2 /* Import WIPO national stage applications for
   applications in our sample */

4 insheet using byhand/wipo_india.csv, comma names clear
  joinby pct_nbr using tmp/TPF_extract_pharma,
    unmatched(both)
6 tab _merge
  /* Keep only those from TPF_extract_pharma which have
    an indian national phase application */
8
  keep if _merge==3
10
  /* Generate a PCT year based on the PCT number, and
    keep pre-2011 only */
12
  gen year = real(substr(pct,3,4))
14 keep if year<=2011
  drop year _merge
16 save tmp/TPF_extract_pharma_india, replace
<</dd_do>>

```

Finally, to keep the sample size tractable (since our coding of applications and outcomes below is done by hand) we focused on applications where July was the

priority month:

```
1 <<dd_do: noout>>
  use tmp/TPF_extract_pharma_india, clear
3 tostring first_prio, replace force
  replace first_prio = substr(first_prio,1,4) + "/" +
    substr(first_prio,5,2) + "/" +
    substr(first_prio,7,.)
5
  gen priodate = date(first_prio,"YMD")
7 format %d priodate

9 gen prioyear=year(priodate)
  gen priomonth = month(priodate)
11 keep if priomonth==7
  save tmp/finalextract, replace
13 <</dd_do>>
```

This left 1,993 PCT applications with an Indian national stage application.

Coding the applications as primary or secondary

We provided the PCT applications to a patent attorney with expertise in drug patents, and asked her to code the claims according to the coding guide described in Sampat and Shadlen (2017), which in turn was adapted from Hemphill and Sampat (2011, 2012). In general we coded each patent as to whether it contained the following types of claims:

- A: active ingredient (see specific descriptions of A1-A4 below)
- B: formulation or composition
- C: method of use
- D: other, but related to the drug
- E: biologic

A patent application can, and often does, include more than one category of claims. We also included a category “Z” for applications that were not actually drug related.

For active ingredient claims, we distinguished the four subcategories:

- A1: active ingredient.
- A2: is for polymorphs or other crystal forms.
- A3: is for enantiomers or other isomers.
- A4: salt, metabolite, or intermediate. Also pre-metabolites and derivatives

The file `byhand/claimscoding.csv` includes coding of each of these applications.

We dropped all applications with only pure product claims (category “D”) or those with only biologic claims (“E”) or non-pharma applications (“Z”). In

practice, the DWPI filtering worked well so there were only a small number of E and Z applications in the set.

We categorized as secondary applications without at least one “A1” chemical compound claim. This is thus a narrow definition of primary applications, i.e. excludes those with polymorph/crystalline structure claims (A2), enantiomer and stereoisomer claims (A3), salts, metabolites, intermediates, pre-metabolites, and derivatives (A4), *unless they also have an A1 claim*.

The resulting file, including is `tmp/finalextract-coded.dta`.

```
<<dd_do: noout>>
2 insheet using byhand/claimscoding.csv, clear comma
  keep if (a1_final + a2_final + a3_final + a4_final +
    b_final + c_final) >0
4 gen secondary = a1_final1==0
  keep pct_nbr secondary
6 joinby pct_nbr using tmp/finalextract,
  unmatched(master)
  assert _merge==3
8 drop _merge
  keep pct_nbr secondary national_number prioyear
10
12 save tmp/finalextract-coded, replace
<</dd_do>>
```

Determining Indian outcomes for the applications

We also searched for the Indian outcomes for each application on the Indian patent office website <<https://ipindiaservices.gov.in/publicsearch>>. The file `byhand/ipo-outcomes.csv` contains status, aggregated to five categories (Abandoned, Granted, Pending, Refused, Withdrawn).

Assessing role of 3(d) from FERs

Next, used the Indian patent office database <<https://ipindiaservices.gov.in/publicsearch>> to locate the first examination reports for the applications. Specifically, we searched the Application Status tab for each application, then used information in the “View Documents” or “View Examination Report” tabs to locate the FER.

For applications that had an FER available, we read the FER and determined if there was (1) any 3(d) objection and (2) a 3(d) objection on claim 1. The file `byhand/fer_coded_3d.csv` contains the coding. For applications with an FER, the field `claim1_3d` is blank if there is no 3(d) objection in the FER, 0 if there

is a specific 3(d) objection but not on claim 1, and X if there is a general 3(d) objection not specific to any claim. As explained in the text, often the FERs are vague, referring generally to “claims” not meeting the standards of Section 3(d), without specifying which claims. These are coded as “X”. However, where FERs refer to all of the claims in an application (e.g. “the claims,” “the present claims,” or “the claims in this invention”) these are coded as 1.”

For 2005-2007 applications with FERs (almost all of which were electronic, making them easier to code) we also coded whether and where there was a novelty or inventive step objection. The file `byhand/fer_coded_3d_detailed.csv` contains these codings. The fields `claim1_novinv` and `anynovinv` are coded analogously to `claim1_3d` above.

For applications from 2005-2007 that were granted we also determined the grant lag based on certificate of issue date and application date (based on information from the Application status tab). These are collected in `byhand/fer_coded_3d_detailed_grants.csv`.

We converted each of the input files generated byhand to Stata, so they could be used in the analyses.

```
<<dd_do: noout>>
2 /* Convert all handed coded files to Stata to merge
   with the final set of coded applications */

4 insheet using byhand/fer_coded_3d.csv, names comma
  clear
  rename inputnational_number national_number
6 save tmp/fer_coded_3d, replace

8 insheet using byhand/fer_coded_3d_detailed.csv, names
  comma clear
  rename inputnational_number national_number
10 save tmp/fer_coded_3d_detailed, replace

12 insheet using
    byhand/fer_coded_3d_detailed_grants.csv, names
    comma clear
  rename inputnational_number national_number
14 save tmp/fer_coded_3d_detailed_grants, replace

16 insheet using byhand/ipo-outcomes.csv, names comma
  clear
  rename inputnational_number national_number
18 save tmp/ipo-outcomes, replace

20 <</dd_do>>
```

Finally, we merged these files containing application outcomes, codings of objection types for applications with FER, coding of detailed objection types for 2005-7 applications with FERs, and information on grant lags for the granted applications from the 2005-7 cohort with the basic application file (`finalextract_coded`). We also added variable and value labels to create the final dataset to be used in the analyses, `processed/india3d.dta`. An CSV file with the same name is also produced for those who wish to reproduce using different software.

```
<<dd_do: noout>>
2 use tmp/finalextract-coded, clear

4 label var pct_nbr          "Application number"
  label var secondary        "Secondary (1=yes)"
6 label var national_number   "Indian application
  number"
  label var prioyear          "Priority year"
8
  /* Merge in outcomes */
10
  joinby national_number using tmp/ipo-outcomes,
    unmatched(master)
12 assert _merge==3
  drop _merge
14
  /* Merge in overall FER coding */
16
  joinby national_number using tmp/fer_coded_3d,
    unmatched(master)
18 assert _merge==3
  drop _merge
20
  gen any3d = claim1_3d~=""
22
  label var anyfer            "Has FER"
24 label var any3d            "Any 3d objection?"

26 /* Merge in 2006-7 detailed FER coding */

28 joinby national_number using
  tmp/fer_coded_3d_detailed, unmatched(master)

30 gen detailedfer = _merge==3
  drop _merge
32
  gen anynovinv = claim1_novinv~=""
```

```

34 replace anynovinv = . if detailedfer==0

36 label var detailedfer "Has detailed coding of FER
    (2006-7 only)"
    label var anynovinv "Any novelty/inventive step
        objection?"

38
    /* Merge in information on grant lags for granted
        applications 2006-7 */
40
    joinby national_number using
        tmp/fer_coded_3d_detailed_grants, unmatched(master)
42 assert _merge==3 if (detailedfer ==1 &
    outcome=="Grant")
    drop _merge
44
    label var lag "Grant lag in days(2006-7 granted
        applications only)"
46
    order pct national_number prioyear secondary outcome
        anyfer claim1_3d any3d detailedfer claim1_novinv
        anyninv lag
48
    label define yn 0 "no" 1 "yes"
50 label values any3d yn
    label values anyninv yn
52
    replace claim1_3d="-9" if claim1_3d==" "
54 replace claim1_novinv = "-9" if claim1_novinv==" " &
        detailedfer==1

56
    replace claim1_3d = "999" if claim1_3d=="X"
58 replace claim1_novinv = "999" if claim1_novinv=="X"

60
    deststring claim1_3d claim1_novinv, replace force
62

64 tab claim1_3d claim1_novinv , row col missing

66 label define rejcat -9 "none" 0 "other claim" 1
    "claim 1" 999 "vague"
    label values claim1_3d rejcat
68 label values claim1_novinv rejcat

```

```

70 label var claim1_3d "3(d) objection type"
   label var claim1_novinv "Novelty/Inventive Step
      objection type"
72
   gen grant = outcome=="Grant"
74 label define grant1 0 "not granted" 1 "granted"
   label values grant grant1
76
   label define secondary1 0 "Primary" 1 "Secondary",
      replace
78 label values secondary secondary1

80 gen year = real(substr(pct,3,4))

82 label var year "Application Year"

84 save processed/india3d, replace
   codebook
86
   outsheet using processed/india3d.csv, names comma
      replace
88

90 <</dd_do>>

```

Analysis

Below we reproduce the full code run using the `india3d` file, to generate the graphs in the text, and also any results related to the graph (e.g. statistical tests) that are noted in the text.

Figure 1

```

<<dd_do>>
2 use processed/india3d

4
   * Discussion in text: Share of applications with FER
      over time
6
   tab anyfer if year>=2001 & year<=2004
8   tab anyfer if year>=2008 & year<=2011

```

```

10 * Discussion in text: How many FER could we find
    count if anyfer==1
12
    * Discussion in text: Share of applications with FER
      by status
14 tab outcome anyfer, row column

16 * Discussion in text: Share with 3d and FER that have
    ambiguous 3(d)

18 tab claim1_3d  if year>=2001 & year<=2004  &
    anyfer==1 & any3d ==1
    tab claim1_3d  if year>=2008 & year<=2011  &
    anyfer==1 & any3d ==1
20

22 /* Focus analysis on applications with FER */

24 keep if anyfer==1

26 /* Create indicators for general and specific 3(d)
    objections */

28 gen rejected3d = any3d==1
    gen rejected3d1 = claim1_3d==1
30
    /* Generate the graph */
32
    collapse (mean) rejected3d rejected3d1, by(year)
34 label var rejected3d "3(d) objection"
    label var rejected3d1 "3(d) objection on claim 1"
36 keep if year>=2002 & year<=2010
    twoway (line rejected3d rejected3d1 year),
        scheme(tufte)
38 <</dd_do>>

```

```

<<dd_graph: saving("figure1.png") alt(" ") replace height(400)>>
<<dd_graph: saving("figure1.eps") alt(" ") replace height(400)>>

```

Figure 2

```

<<dd_do>>
2 use processed/india3d,clear
  keep if year>=2006 & year<=2007
4 keep if anyfer==1
  assert detailedfer==1

```

```

6      * Discussion in text
8
9      tab any3d anynovinv, row column cell
10
11     /* Generate the graph */
12
13     tabplot any3d anynovinv , scheme(tufte) showval
14
15     <</dd_do>>

```

<<dd_graph: saving("figure2.png") alt(" ") replace height(400)>>
 <<dd_graph: saving("figure2.eps") alt(" ") replace height(400)>>

Figure 3

```

1 <<dd_do>>
2
3 use processed/india3d,clear
4 keep if detailedfer==1
5
6     * In text discussion
7     tab claim1_3d claim1_novinv , row column cell
8
9     /* Generate the graph */
10
11     tabplot claim1_3d claim1_novinv , scheme(tufte)
12         showval
13
14 <</dd_do>>

```

<<dd_graph: saving("figure3.png") alt(" ") replace height(400)>>
 <<dd_graph: saving("figure3.eps") alt(" ") replace height(400)>>

Figure 4

```

1 <<dd_do>>
2
3 use processed/india3d,clear
4 keep if detailedfer==1
5
6     /* Generate categories for nov/inv plus 3(d) vs.
7         none*/
8
9     gen cat1 = .
10    replace cat1 = 0 if any3d==0 & anynovinv==0

```

```

11 replace cat1 = 1 if any3d==1 & anynovinv==1
12
13 * In text discussion of grant rates by categories
14
15 tab cat1, sum(grant)
16 ttest grant, by(cat1)
17
18 /* Generate categories for nov/inv alone vs. with
19    3(d) */
20
21 gen cat2 = .
22 replace cat2 = 0 if any3d==0 & anynovinv==1
23 replace cat2 = 1 if any3d==1 & anynovinv==1
24
25 * In text discussion of grant rates by categories
26
27 tab cat2, sum(grant)
28 ttest grant, by(cat2)
29
30 /* Collapse and graph */
31
32 gen count = 1
33 collapse (mean) grant (sum) count, by(any3d anynovinv)
34
35 decode any3d, gen(any3dl)
36 decode anynovinv, gen(anynovinvl)
37
38 gen cat = "3(d): " + any3dl + "; " + "nov/inv: " +
39    anynovinvl + " (N=" + string(count) + ")"
40
41 graph hbar (asis) grant, over(cat, sort(count)
42    descending) blabel(bar, format(%3.2g))
43    ytitle(grant rate) scheme(tufte)
44
45 <</dd_do>>

```

```

<<dd_graph: saving("figure4.png") alt(" ") replace height(400)>>
<<dd_graph: saving("figure4.eps") alt(" ") replace height(400)>>

```

Figure 5

```

<<dd_do>>
2
3 use processed/india3d,clear
4 keep if detailedfer==1

```

```

6  /* Generate categories for only nov/inv on claim 1
   vs. also 3d on claim 1 */

8  gen cat1=.
   replace cat1=0 if claim1_3d== -9 & claim1_novinv==1
10 replace cat1=1 if claim1_3d==1 & claim1_novinv==1

12 * In text discussion of differences between categories
   tab cat1, sum(grant)
14 ttest grant, by(cat1)

16 /* Collapse and graph */

18 gen count = 1

20 collapse (mean) grant (sum) count, by(claim1_3d
   claim1_novinv)

22 decode claim1_3d, gen(claim1_3dl)
   decode claim1_novinv, gen(claim1_novinvl)
24
   gen cat = "3(d): " + claim1_3dl + "; " + "nov/inv:
   " + claim1_novinvl + " (N=" + string(count) + ")"

26 graph hbar (asis) grant, over(cat, sort(count)
   descending) blabel(bar, format(%3.2g))
   ytitle(grant rate) scheme(tufte)

28

30 <</dd_do>>

```

```

<<dd_graph: saving("figure5.png") alt(" ") replace height(400)>>
<<dd_graph: saving("figure5.eps") alt(" ") replace height(400)>> ##
Figure 6

```

```

<<dd_do>>

2  use processed/india3d,clear
4  keep if detailedfer==1 & grant==1

6  * In text discussion of lags for applications with
   and without 3(d) objections

8  ttest lag, by(any3d)

10 /* Generate categories for nov/inv with and without

```

```

3(d) */
12 gen cat1=.
   replace cat1=1 if any3d==1 & anynovinv==1
14 replace cat1=0 if any3d==0 & anynovinv==1

16 * In text discussion of lags by novelty/inventive
   step only and novelty/inventive step plus 3(d)

18 ttest lag, by(cat1)

20 /* Collapse and graph */

22 gen count = 1
   collapse (mean) lag (sum) count, by(any3d anynovinv)
24
   decode any3d, gen(any3dl)
26 decode anynovinv, gen(anynovinvl)

28 gen cat = "3(d):" + any3dl + "; " + "nov/inv:" +
   anynovinvl + " (N=" + string(count) + ")"

30 graph hbar (asis) lag, over(cat, sort(count)
   descending) blabel(bar, format(%4.1f))
   ytitle(days) scheme(tufte)

32 <</dd_do>>

```

```

<<dd_graph: saving("figure6.png") alt(" ") replace height(400)>>
<<dd_graph: saving("figure6.eps") alt(" ") replace height(400)>>

```

Figure 7

```

<<dd_do>>
2
   use processed/india3d,clear
4 keep if anyfer==1

6 /* Generate a variable for claim 1 specific 3d
   objection */

8 gen claim1_3d_specific = claim1_3d == 1

10 * In text discussion of primary vs. secondary

12 tab secondary

```

```

14  * In text discussion of primary vs. secondary vs. 3(d)
16  tab secondary claim1_3d
18  * In text discussion of claim1 specific vs. secondary
20  tab secondary, sum(claim1_3d_specific)
22  ttest claim1_3d_specific, by(secondary)

24  /* Generate the figure */

26  tabplot claim1_3d secondary , scheme(tufte) showval

28  * In text discussion of any 3(d) vs. secondary

30  tab secondary any3d, row column
32  ttest any3d, by(secondary)

34  <</dd_do>>

```

```

<<dd_graph: saving("figure7.png") alt(" ") replace height(400)>>
<<dd_graph: saving("figure7.eps") alt(" ") replace height(400)>>

```

Figure 8

```

<<dd_do>>

2  use processed/india3d,clear
4  keep if anyfer==1

6  gen claim1_3d_specific = claim1_3d == 1

8  * Discussion in text: 3d for most recent applications

10 tab any3d if year >=2009 & year <=2011
12 tab claim1_3d_specific if year >=2009 & year <=2011

14 gen rejected3d1 = claim1_3d==1

16 /* Collapse and graph */

18 collapse (mean) any3d rejected3d1, by(year secondary)
label var any3d "3(d) objection"

```

```

    label var rejected3d1 "3(d) objection on claim 1"
20 label var year "Application Year"

22 keep if year>=2002 & year<=2010

24 label define secondary1 0 "Primary" 1 "Secondary",
    replace
    label values secondary secondary1
26

28 twoway (line any3d rejected3d1 year), scheme(tufte)
    by(secondary)

30 <</dd_do>>

```

```

<<dd_graph: saving("figure8.png") alt(" ") replace height(400)>>
<<dd_graph: saving("figure8.eps") alt(" ") replace height(400)>>

```

Clean up

This will delete intermediate files to save space.

```

<<dd_do: nooutput>>
2 foreach x in TPF_Core TPF_IPC TPF_PCT
    TPF_extract_pharma_india family_to_ipc
    fer_coded_3d fer_coded_3d_detailed
    fer_coded_3d_detailed_grant finalextract-coded
    finalextract ipo-outcomes
    fer_coded_3d_detailed_grants TPF_extract
    TPF_extract_pharma {
capture erase tmp/`x'.dta
4 }
<</dd_do>>

```

Generate a PDF version of the code

If you have Pandoc installed, the following command should generate the current PDF file which is just a slightly better formatted version of the Markdown file: `pandoc "code/Data Appendix.md" --listings -H code/listings-setup.tex -o "Data Appendix.pdf"`

References

Hemphill, C. Scott, and Bhaven N. Sampat. “Evergreening, patent challenges, and effective market life in pharmaceuticals.” *Journal of Health Economics* 31.2 (2012): 327-339.

Hemphill, C. Scott, and Bhaven N. Sampat. “When do generics challenge drug patents?.” *Journal of Empirical Legal Studies* 8.4 (2011): 613-649.

Sampat, Bhaven N., and Kenneth C. Shadlen. “Secondary pharmaceutical patenting: A global perspective.” *Research Policy* 46.3 (2017): 693-707.
